# Bilingualism and creativity across development: Evidence from divergent thinking and convergent thinking

**DOI:** 10.3389/fnhum.2022.1058803

**Published:** 2023-01-06

**Authors:** Yifan Zheng, Solange Denervaud, Stephanie Durrleman

**Affiliations:** ^1^Department of Neuroscience and Movement Science, Faculty of Science and Medicine, University of Fribourg, Fribourg, Switzerland; ^2^Department of Neurology, Guangdong Provincial Key Laboratory of Diagnosis and Treatment of Major Neurological Diseases, National Key Clinical Department and Key Discipline of Neurology, The First Affiliated Hospital, Sun Yat-sen University, Guangzhou, China; ^3^Department of Diagnostic and Interventional Radiology, Lausanne University Hospital (CHUV), University of Lausanne (UNIL), Lausanne, Switzerland

**Keywords:** bilingualism, divergent thinking, convergent thinking, gray matter volume, cortical thickness, children and adolescents

## Abstract

**Introduction:**

Numerous studies have demonstrated the benefits of creativity from bilingualism. Divergent thinking and convergent thinking are considered the two most important components of creativity. Various (although not all) studies have concluded that bilingual children outperform monolingual children in divergent thinking, however, no study on children or adolescents so far has explored the relation between bilingualism and convergent thinking, or the brain structural basis of interaction between bilingualism and creativity. This study aimed to explore the impact of bilingualism on both convergent and divergent thinking in children and adolescents based on neuropsychological assessments, and the possible structural basis of the effect of bilingualism on creativity by a whole-brain analysis of regional gray matter volume (rGMV) and cortical thickness in children and adolescents.

**Methods:**

92 healthy children and adolescents of age 4–18 were recruited from public or private schools in the French-speaking side of Switzerland. Demographic data of the participants were collected, including gender, age, pedagogy, usage of language, and parents’ socioeconomic status. Most of the participants underwent the neuropsychological assessments of divergent thinking, convergent thinking, and fluid intelligence. Structural image data of 75 participants were analyzed. Both voxel-based morphometry (VBM) and surface-based morphometry (SBM) were processed, to perform the analyses of rGMV and cortical thickness respectively.

**Results:**

The outcomes indicated that convergent thinking, but not divergent thinking benefits from bilingualism in children and adolescents. However, this bilingual advantage appears to weaken across development. Unexpectedly, no significant correlation between morphometry and bilingualism was found. Neither divergent thinking scores nor convergent thinking scores showed any significant correlation with rGMV. However, the whole brain SBM showed that the cortical thickness in the right supplementary motor area (SMA) was negatively correlated with convergent thinking scores, which suggested that the children and adolescents with higher convergent thinking abilities may have thinner, more mature, and more activated cortex in the right SMA.

**Discussion:**

Bilingualism and cortical thinness in the right SMA might facilitate convergent thinking independently, by enhancing this selective ability.

## 1. Introduction

Increasing evidence suggests that there are several cognitive benefits that come from bilingualism, including creativity (Kharkhurin, 2009; Hommel et al., 2011; Leikin and Tovli, 2014; Sampedro and Peña, 2019). Creativity, a challenging concept to define, generally refers to the ability to produce something useful and original (Zhu et al., 2013), and it is known to promote progress and innovation in human societies (Dietrich and Kanso, 2010). In the last five decades, numerous studies have demonstrated an advantage of creativity in plurilingual people (Torrance et al., 1970; Carringer, 1974; Okoh, 1980; Karapetsas and Andreou, 1999; Kharkhurin, 2008, 2009). The interpretation of this advantage includes two hypotheses. Firstly, switching between two languages can enhance executive functions (Bialystok, 2015), which subsequently could enhance creative thinking (Lee and Therriault, 2013). Secondly, the more diverse life experiences of bilinguals may in turn make them more creative (Ritter et al., 2012).

Divergent thinking and convergent thinking are considered the two most important components of creativity (Guilford, 1967). On the one hand, divergent thinking can be defined as the process that allows people to generate a wide variety of outputs in a novel way, based on relatively weak constraints (Hommel et al., 2011; Zhao et al., 2019). On the other hand, convergent thinking can be defined as a more strongly constrained process in which people combine information and search for the best outcome (Hommel et al., 2011; Zhao et al., 2019).

There are abundant studies exploring the relation between bilingualism and divergent thinking, and various conclude that bilingual children and adolescents outperform monolingual children and adolescents on this skill (Torrance et al., 1970; Carringer, 1974; Okoh, 1980). Nevertheless, some studies have given rise to null or even opposite results (Rosselli et al., 2000; Gollan et al., 2002; Lange et al., 2020; Booton et al., 2021). For instance, the study by Booton et al. (2021) did not find any significant difference between monolingual and bilingual children in three measures of divergent thinking. Studies by Rosselli et al. (2000) and Gollan et al. (2002) even found that monolinguals outperformed bilinguals in a verbal fluency task which was similar to the divergent-thinking task.

In contrast to the body of work on divergent thinking in bilinguals, only a few studies have focused on the relation between bilingualism and convergent thinking. Benefits of convergent thinking from bilingualism have been found in studies on adults (Hommel et al., 2011; Zhao et al., 2019), however, no study on children and adolescents so far has explored the relation between bilingualism and convergent thinking. Furthermore, neuroimaging studies on bilingualism in children and adolescents are limited. One of them has demonstrated that increases in gray matter volume (GMV) of the left lower parietal region are associated with improved behavioral performance in attentional control in multilingual children (Della Rosa et al., 2013). However, to the best of our knowledge, no study has been concerned with the brain structural basis of interaction between bilingualism and creativity in children and adolescents. In light of findings in adults indicating that creativity is associated with GMV of widely distributed cortical and subcortical regions (i.e., the left rostrolateral prefrontal cortex, the left inferior parietal lobule, the right insula, the right dorsolateral prefrontal cortex, the right parietal lobe, the middle cingulate cortex/precuneus, bilateral striata, the left cerebellum crus 1, and the right supplementary motor area (Takeuchi et al., 2010; Gansler et al., 2011; Jung et al., 2013; Bendetowicz et al., 2017; Ogawa et al., 2018), it seems relevant to investigate whether morphometric data like regional gray matter volume (rGMV) or cortical thickness are associated with the impact of bilingualism on creativity in children and adolescents.

The current study aimed to explore bilingualism effects on both convergent and divergent thinking based on neuropsychological assessments while controlling for possible confounds. One of these confounds is fluid intelligence, as Kharkhurin (2009) found that Farsi–English bilingual college students had higher fluid intelligence than their Farsi monolingual pairs, and intelligence, in turn, has been shown to predict creativity by some studies (Kim, 2005; Silvia, 2008). Other studies, however, have indicated that intelligence and creativity are only modestly related to each other (Batey and Furnham, 2006). Even though the relation between intelligence and creativity is controversial, it nevertheless makes sense to control for intelligence as a potential confound in a study on the relationship between bilingualism and creativity. Another potential confounding factor to remain attentive to in the current work is the socioeconomic status (SES) of the family, as this may also play a crucial role in creativity. Indeed, Jankowska and Karwowski revealed that children’s initial level of creative thinking is related to their family’s SES (Jankowska and Karwowski, 2019).

In sum, previous studies examining the relation between bilingualism and divergent thinking have yielded mixed results and the potential effects of bilingualism on convergent thinking have scarcely been examined, particularly in children and adolescents. This work thus seeks to contribute to our understanding of whether the effects of bilingualism on divergent thinking are robust, and whether these effects also carry over to convergent thinking. In addition, the impact of bilingualism on divergent and convergent thinking has been under-investigated in children and adolescents, although the cognitive outcomes of bilingualism have been claimed to be more pronounced in this group (as well as older adults), than in young adults for other abilities other than creativity, e.g., executive functions (Bialystok et al., 2005; Chung-Fat-Yim et al., 2019). By targeting children and adolescents in this work, therefore, we aimed to contribute to filling the gaps in our understanding of the lifetime trajectory of effects on creativity (both divergent and convergent thinking), that yield contradictory or scarce results, and are particularly understudied in children and adolescents for whom such effects may be more likely to emerge. In addition, we explore the relations between rGMV/cortical thickness and bilingualism, as well as the relations between rGMV/cortical thickness and divergent/convergent thinking, by a whole-brain analysis of rGMV and cortical thickness in structural magnetic resonance imaging (sMRI), in order to identify a possible structural basis of the effect of bilingualism on creativity.

## 2. Materials and methods

### 2.1. Participants

A total of 92 healthy participants including 85 children and 7 adolescents were recruited as part of a large study investigating the effect of pedagogy on child development. Both children and adolescents were recruited in schools in the French-speaking side of Switzerland. As they were attending either public (60 participants) or private (Montessori) schools (32 participants), we ensured that the proportion of participants from each pedagogical system was equal among bilingual and monolingual groups. The inclusion criterion was thus to be of school age range, i.e., 4 to 18 years. The study excluded children and adolescents with learning disabilities, sensory or motor impairments reported by their parents.

This study was performed in line with the principles of the Declaration of Helsinki. The study protocol was approved by the local Ethics Committee (CER-Vaud). Written and oral consents were obtained from parents and participants respectively.

### 2.2. Demographic data

Demographic data of the participants were collected, including gender, age, pedagogy, usage of language, and parents’ SES. Usage of language was assessed based on parental reports, answering the question “Do you and your child speak another language at home?,” if yes, then the language(s) were enumerated. SES was assessed through a parental questionnaire, in which parents were asked to provide information about their educational and professional levels (Genoud, 2011). The answer given by each parent was rated from 1 to 4. When the participant was under biparental authority, the average score of both parents was computed. In the case of uniparental authority, the score was based on the answer from the legal guardian. Scores were normalized to range from 0 to 1, with 0 denoting low SES, while 1 reflects high SES.

### 2.3. Neuropsychological assessments

The participants underwent a series of neuropsychological assessments of divergent thinking, convergent thinking, and fluid intelligence^1^.

#### 2.3.1. Divergent thinking task

The participants were instructed to draw as many different drawings as possible from an abstract shape within a predefined time of 5 min. They were asked to be as creative as possible. The sum of each valid drawing, which referred to a concrete and unique drawing using the initially given shape, was applied to rate divergent thinking. The rating had no maximum (Lubart et al., 2011).

#### 2.3.2. Convergent thinking task

A standardized drawing task, designed and calibrated for children, was used to assess convergent thinking. In this task, each participant was asked to draw one picture on a sheet of paper by including at least 3 of the 8 abstract shapes as creatively as possible within a time limit of 10 min. An abstract shape could be an oval, a square, a triangle, and so on. The final drawing was rated from 1 (drawing with low convergent thinking ability) to 7 (drawing with high convergent thinking ability). The criteria of the rating included the integration of abstract shapes, originality, and storytelling achievement of the drawing. The rating was completed by three trained raters independently. The final score was computed by taking the average of the scores from the three raters (Lubart et al., 2011).

#### 2.3.3. Fluid intelligence (PM-47)

Fluid intelligence was measured through a black-and-white version of the Raven’s Progressive Matrices (PM-47) test (Capirci et al., 1998), composed of 36 incomplete matrices. For each matrix, the participants were presented with six possible patterns and asked to select one to complete the missing part. The time limit was set to 15 min, which allowed enough time to go through all the matrices, independently of age. To score fluid intelligence, correct answers were summed (score range: 0 to 36).

### 2.4. Statistical analyses

The R-based Jamovi Freeware (version 2.0.0.0) was used to perform the statistical analyses. Multiple independent sample *t*-tests were performed to determine the differences in age, SES, fluid intelligence, and creativity scores between the bilingual group and the monolingual group. χ^2^ tests were performed to compare categorical data (gender ratio, pedagogical background) between these two groups. Analyses of covariance (ANCOVA) were performed to explore factors correlated with divergent thinking scores or convergent thinking scores respectively. Independent factors having a possible association with divergent thinking score, or convergent thinking score were entered into the model (bilingualism, SES, fluid intelligence, pedagogy), and age as a covariate, plus interaction terms (bilingualism × age, pedagogy × age). Results with *p* < 0.05 were considered statistically significant.

### 2.5. Image acquisition, preprocessing, and data analysis

#### 2.5.1. MRI acquisition protocol

Structural MRI data were acquired in 92 participants, who matched the safety criteria required for MRI, at the Lemanic Biomedical Imaging Center (CIBM) of the University Hospital Lausanne (UNIL-CHUV) on a Siemens 3T Magnetom Prisma equipped with a 64-channel head coil. For each participant, a magnetization-prepared rapid acquisition gradient echo (MPRAGE) was applied to obtain a 3-dimensional high-resolution isotropic T1-weighted (T1w) image of the whole brain. The MPRAGE-T1w images were acquired with parameters as follows: echo time (TE) = 2.47 ms, repetition time (TR) = 2,000 ms, inversion time (TI) = 900 ms, flip angle (FA) = 8*^o^*, field of view (FOV) = 160 × 240 × 256 mm^3^ and voxel size = 1 × 1 × 1 mm^3^.

Images acquired from 17 participants were excluded because of motion artifacts during the MRI acquisition (*n* = 7), dental braces interference (*n* = 7), or outside target-age (*n* = 3). Finally, image data from 75 participants were included for preprocessing and morphometric analyses.

#### 2.5.2. Image preprocessing and morphometric analysis

Images of 75 participants were preprocessed and analyzed by the CAT12 toolbox (Gaser, Structural Brain Mapping Group, Jena University Hospital, Jena, Germany^2^) working in SPM12 (Wellcome Trust center for Neuroimaging, London, UK^3^) running on MATLAB R2018b (MathWorks, Natick, MA, USA). CAT12 served as the platform for processing both voxel-based morphometry (VBM) and surface-based morphometry (SBM), to perform the analyses of rGMV and cortical thickness. Because there were only 64 participants with convergent thinking scores and 56 participants with divergent thinking scores, correlational analyses between morphometric and convergent/divergent thinking scores were performed respectively.

##### 2.5.2.1. Analysis of rGMV by VBM

The T1 images were normalized to the Montreal Neurological Institute (MNI) template. Afterward, the whole brain images were segmented into gray matter (GM), white matter (WM), and cerebrospinal fluid (CSF). The total intracranial volumes (TIV) were also estimated for each participant to correct for different head sizes and volumes. Next, the resulting images were checked for homogeneity. Finally, the modulated normalized GM images were smoothed using a kernel with 8 mm full-width half-maximum (FWHM), and ready for statistical analysis.

We performed statistical analysis of rGMV by multiple regression in the CAT12/SPM12 statistical module. Taking bilingualism, age, gender, pedagogy and TIV as covariates (convergent thinking and fluid intelligence scores were added to covariates for 64 participants while divergent thinking and fluid intelligence scores were added for 56 participants), we tested the correlations between rGMV and the variables of interest (bilingualism, convergent thinking scores and divergent thinking scores), applying a significant threshold of *p* < 0.05 with Family-wise error (FWE) correction.

##### 2.5.2.2. Analysis of cortical thickness by SBM

We used projection-based thickness to estimate cortical thickness and to create the central cortical surface for the left and right hemispheres (Dahnke et al., 2013). The surface data were resampled and smoothed using a 15 mm kernel as smoothing filter size in FWHM. The quality of surface data was checked by checking sample homogeneity.

We performed statistical analysis of cortical thickness applying multiple regression in the CAT12/SPM12 statistical module. Taking bilingualism, age, gender, and pedagogy as covariates (convergent thinking and fluid intelligence scores were included in covariates for 64 participants while divergent thinking and fluid intelligence scores were included for 56 participants), we tested the correlations between cortical thickness and the variables of interest (bilingualism, convergent thinking scores and divergent thinking scores), applying a significant threshold of *p* < 0.05 with FWE correction.

## 3. Results

### 3.1. Demographic characteristics and neuropsychological assessments of bilingual and monolingual groups

The demographic data and neuropsychological scores were compared between bilingual and monolingual participants. There was no significant difference found in any variable between the two groups (all *p*-values > 0.05, Table 1).

**TABLE 1:** Comparison of demographic data and neuropsychological scores between bilingual and monolingual groups.

	Monolinguals (*n* = 56)	Bilinguals (*n* = 36)	*P-*values	χ^2^/*t* values
Gender (M/F)	26/30	12/24	0.213	1.55
Age (years)	8.81 ± 2.94	8.32 ± 2.76	0.422	0.807
Pedagogy (P/M)	40/16	20/16	0.119	2.43
Parents’ SES	2.96 ± 0.52^a^	3.20 ± 0.61^b^	0.058	−1.921
Divergent score	6.68 ± 3.38^c^	7.43 ± 3.57^d^	0.399	−0.850
Convergent score	3.74 ± 1.66^e^	3.97 ± 1.80^f^	0.559	−0.587
Fluid intelligence	31.9 ± 4.12^g^	31.1 ± 4.48^h^	0.424	0.804

The values are mean ± SD. M, male; F, female; P, public school; M, Montessori school (private school).

^a^3 participants missed.

^b^4 participants missed.

^c^12 participants missed.

^d^13 participants missed.

^e^8 participants missed.

^f^4 participants missed.

^g^8 participants missed.

^h^5 participants missed.

### 3.2. Factors associated with divergent thinking and convergent thinking of children and adolescents

To determine the factors independently associated with divergent thinking scores, ANCOVA was performed on 67 participants (N.B. There were 25 participants missing divergent thinking assessments). As shown in Table 2, neither bilingualism nor any other variable was associated with the divergent thinking score.

**TABLE 2:** Factors associated with divergent thinking score [Analyses of covariance (ANCOVA)].

	Sum of squares	*df*	Mean square	*p*
Age	0.0000			
Bilingualism	0.0691	1	0.0691	0.938
Pedagogy	1.6768	2	0.8384	0.927
SES	0.1400	1	0.1400	0.911
Fluid intelligence	6.6636	1	6.6636	0.445
Bilingualism × Age	0.0216	1	0.0216	0.965
Pedagogy × Age	1.2822	2	0.6411	0.944
Residuals	219.8579	20	10.9929	

Singular fit encountered; one or more predictor variables are a linear combination of other predictor variables.

**FIGURE 1:**
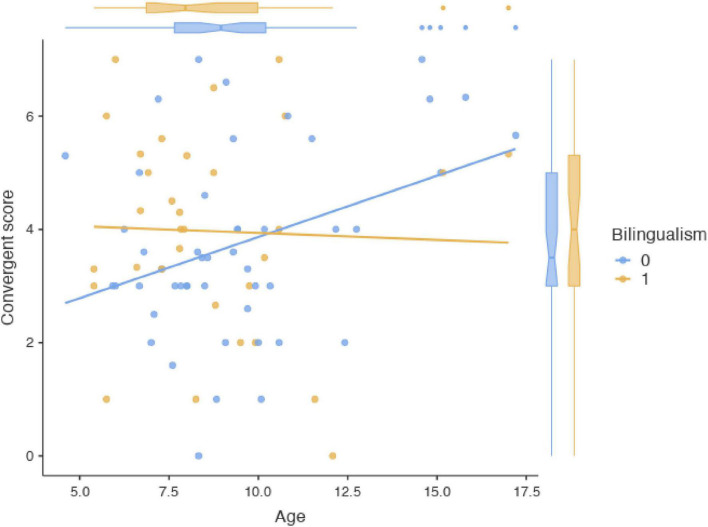
Age adjusted the association between bilingualism and the convergent thinking score.

Analyses of covariance (ANCOVA) was performed in the 80 participants with convergent thinking scores (N.B. There were 12 participants missing convergent thinking assessments). As shown in Table 3, bilingualism was significantly correlated with the convergent thinking score (*p* = 0.006). Additionally, age adjusted the association between bilingualism and the convergent thinking score (bilingualism*age, *p* = 0.006). As shown in Figure 1, bilingualism has a beneficial effect on the convergent thinking score, but this benefit becomes weaker as the age increases and finally, monolinguals present higher convergent thinking scores. We further computed similar statistical analyses on the subset of 67 participants presenting both creative thinking scores (mean age = 9.16, SD = 2.65). Similar effects were observed, with a main effect of bilingualism [F(57,1) = 10.24, *p* = 0.002], as well as an interaction between bilingualism and age [F(57,1) = 10.55, *p* = 0.002]. Bilingual children scored high from early years on, and kept stable convergent thinking abilities across development, while monolingual children scored lower in early years but presented a linear increase across development. No other factors were significantly related to convergent thinking (all *p* > 0.398; see Supplementary Table 1).

**TABLE 3:** Factors associated with convergent thinking score [Analyses of covariance (ANCOVA)].

	Sum of squares	*df*	Mean square	*p*
Age	0.195	1	0.195	0.773
Bilingualism	**18.515**	**1**	**18.515**	0.006*
Pedagogy	0.252	1	0.252	0.743
SES	1.161	1	1.161	0.483
Fluid intelligence	1.431	1	1.431	0.436
Bilingualism × Age	**19.150**	**1**	**19.150**	0.006*
Pedagogy × Age	1.064	1	1.064	0.502
Residuals	158.725	68	2.334	

Asterisks refer to *P* < 0.05, which indicates significant associations between the variables and the convergent thinking score. Bilingualism was significantly correlated with the convergent thinking score (*p* = 0.006). Age adjusted the association between bilingualism and the convergent thinking score (bilingualism × age, p = 0.006). Significant factors are highlighted in bold.

### 3.3. Correlational analysis between morphometric measures and bilingualism

Based on multiple regression analysis of the whole brain VBM, there was no significant correlation between any rGMV and bilingualism.

On the other hand, a multiple regression analysis of the whole brain SBM did not reveal any correlation between cortical thickness and bilingualism.

### 3.4. Correlational analysis between morphometric and neuropsychological measures

#### 3.4.1. Correlation between morphometric measures and divergent thinking score

Taking divergent thinking scores and fluid intelligence as covariates together with bilingualism, age, gender, and TIV, a multiple regression analysis of the whole brain VBM was performed in 56 participants with divergent thinking scores. The result indicated that there was no significant correlation between rGMV and divergent thinking scores.

With divergent thinking scores, fluid intelligence, bilingualism, age, and gender entering the multiple regression model, the whole brain SBM was analyzed in these 56 participants and did not reveal any correlation between cortical thickness and divergent thinking scores.

#### 3.4.2. Correlation between morphometric measures and convergent thinking score

Taking the convergent thinking score, fluid intelligence, bilingualism, age, gender and TIV as covariates, a multiple regression analysis of the whole brain VBM was conducted in 64 participants with convergent thinking scores. As a result, convergent thinking score did not show any significant correlation with rGMV.

**FIGURE 2:**
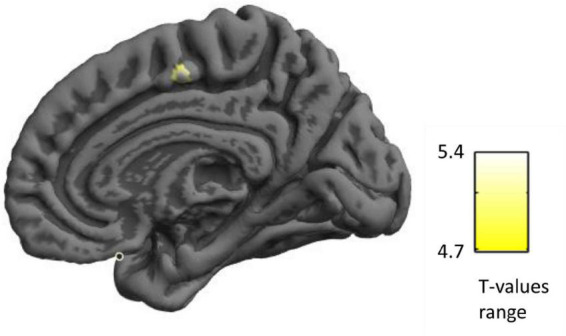
Correlation between cortical thickness and convergent thinking scores: Cortical thickness was negatively correlated with convergent thinking scores in the right supplementary motor area (SMA), which is in yellow.

Surprisingly, a multiple regression analysis of the whole brain SBM in these 64 participants revealed that, after correcting for confounding variables including fluid intelligence, bilingualism, age and gender, the right supplementary motor cortical thickness was negatively correlated with the convergent thinking scores (*p* = 0.005 with FWE correction based on cluster level, cluster size = 18 voxels; *p* = 0.008 with FWE correction based on peak level, *T* = 5.34; see Figure 2 and Table 4), which means that a thinner right supplementary motor cortex may correspond to higher convergent thinking ability.

**TABLE 4:** Brain region with significantly negative correlation between cortical thickness and the convergent thinking scores.

Brain region	MNI peak coordinates			Cluster size	*T*
	*x*	*y*	*z*		
Right SMA	7	−9	55	18	5.34*

*p < 0.05 with FWE correction.

## 4. Discussion

While the relation between bilingualism and creativity in adults has been widely explored at the behavioral and neural levels, less is known about children and adolescents. Here, we investigated whether divergent and convergent thinking measures were related to bilingualism across development in 92 mono-and bilingual children and adolescents. Although no differences between the bilingual and the monolingual groups were observed in terms of divergent and convergent thinking, developmental patterns differed. Bilingual participants showed higher convergent thinking abilities at a younger age, however, this advantage appeared weaker across development. Conversely, monolingual participants presented first lower convergent thinking abilities, while these abilities were higher at an older age. To the best of our knowledge, our study is the first one to explore the relation between bilingualism and convergent thinking in children and adolescents, making the interpretation challenging. It may be that early bilingual abilities allow children to reinforce their higher order abilities (Kushalnagar et al., 2010), enhancing their convergent thinking skills at first as compared to their monolingual peers while this impact lessens with time. Nevertheless, it is hasty to conclude that a decreasing impact of bilingualism systematically arises, since previous studies on adults have found a beneficial effect of bilingualism on convergent thinking in adulthood as well (Hommel et al., 2011; Zhao et al., 2019). Indeed, our study, being cross-sectional, the different ages represent different individuals, thus it is difficult to conclude whether the impact of bilingualism on convergent thinking indeed fluctuates throughout the developmental phases of a single individual or not. Hence, longitudinal studies would be useful to shed light on the potential long-term effects of bilingualism on convergent thinking.

In contrast to the effect observed for convergent thinking, no developmental effect of bilingualism could be detected on divergent thinking in our sample. This outcome is consistent with a recent report of no bilingual advantage in divergent thinking amongst children in three measures of divergent thinking (Booton et al., 2021). Owing to the larger samples and more comprehensive consideration of potential confounds, this report of Booton’s seems more reliable and valid than the previous studies producing mixed results (reviewed by Kharkhurin, 2011). Given the bilingual benefits on convergent thinking but not divergent thinking, the current findings are partially consistent with the results of the study by Hommel et al. (2011), which concluded that high-proficient bilinguals outperformed low-proficient bilinguals in convergent thinking, but not in divergent thinking. As they suggest, it appears that bilingualism leads to a relatively focused cognitive-control state characterized by more constraints for solutions and stronger local competition for selection, which fits well with the cognitive control requirements of convergent thinking, but not divergent thinking (Hommel et al., 2011).

In addition to investigating behavioral repercussions of bilingualism on creative thinking, this study also explored possible structural bases of these behavioral findings, using morphometric approaches. Unexpectedly, no notable correlation between rGMV and bilingualism was revealed by a multiple regression analysis of the whole brain VBM, after correcting for gender, age and TIV. Meanwhile, a null correlation between cortical thickness and bilingualism was identified by multiple regression analysis of the whole brain SBM. Neuroimaging research on bilingualism in children or adolescents are rare, with so far only one study investigating the relation between bilingualism and rGMV in children and reporting greater GMV in eight clusters and less GMV in five clusters in early bilingual children than monolingual children. The eight brain regions with greater GMV included left precentral gyrus, left cerebellum lobule VIII, right inferior orbitofrontal gyrus, right para-hippocampal gyrus, right supplementary motor area, right cerebellum lobule VIII, right postcentral gyrus, and right precuneus, while the five regions with less GMV consisted of left gyrus rectus, left supramarginal gyrus, left angular gyrus, left superior occipital gyrus, and right cerebellum lobule IX (Schug et al., 2022). Our null findings may stem from the fact that our sample lumped together both early bilinguals and late bilinguals. Future work should keep track of age of acquisition of the second language and proficiency levels to ascertain whether indeed structural effects of bilingualism related to creativity may arise for the early bilinguals or the more proficient (balanced) bilinguals [for effects of these factors on other domains see Cargnelutti et al. (2019); Claussenius-Kalman et al. (2020); Liu et al. (2021)].

In previous studies, creativity appeared to be associated with both increases and decreases in rGMV and/or cortical thickness across broad brain regions (Takeuchi et al., 2010; Gansler et al., 2011; Jung et al., 2013; Bendetowicz et al., 2017; Ogawa et al., 2018). In our study, neither divergent thinking scores nor convergent thinking scores showed any significant correlation with rGMV. However, the whole brain SBM revealed that convergent thinking scores were negatively correlated with the right supplementary motor cortical thickness, which was the single significant correlation to emerge from the morphometric data. Decreased cortical thickness in children has been demonstrated by previous studies to be associated with more mature brain cortex (Tamnes et al., 2010) and increased brain activation from fMRI (Nunez et al., 2011). Thus, our result suggests that the participants with higher convergent thinking abilities may have thinner, more mature, and more activated cortex in the right supplementary motor area (SMA). SMA is localized in the posterior part of the superior frontal gyrus (Penfield and Welch, 1951) and is part of the motor regions which play a role in action planning, appropriate behavior selecting and deciding (Berkowitz and Ansari, 2008; Zhu et al., 2016). These cognitive activities are involved in the convergent thinking task in our study. A previous neuroimaging study by Fang Tian et al. on visual creativity in healthy adults also observed that visual creativity was significantly negatively correlated with cortical thickness in right SMA, as well as some other brain regions (Tian et al., 2018). The task of visual creativity in their study was supposed to have demands for the above cognitive processes too. Interestingly, the association between convergent thinking scores and rGMV of right SMA was not detected by the whole brain VBM. This inconsistency might be because the sensitivity and accuracy of cortical thickness are higher than rGMV (Fischl and Dale, 2000; Kotini et al., 2004). In this study, both creativity tasks were drawing-based tasks. It would be interesting to test whether this effect would be observed with a verbal convergent thinking task. It could be specifically related to motor-based creative thinking activities.

Moreover, the reason for the null correlations between divergent thinking scores and morphometric measures in the current work might be due to the different requirements for action planning, appropriate behavior selecting and deciding between divergent thinking and convergent thinking. The selection criteria for divergent thinking are relatively vague and the decision of the solution can be more flexible. In contrast, convergent thinking only allows one solution to a well-defined problem, which has more demands for action planning, selecting, and deciding (Hommel et al., 2011; Zhao et al., 2019).

Because no correlation between bilingualism and morphometric measures was found in our study, the cortical thinness in right SMA should not be interpreted as the structural basis of the impact of bilingualism on convergent thinking. Instead, we conjecture that bilingualism and cortical thinness in right SMA might facilitate convergent thinking independently. Interestingly, the underlying mechanisms of how these two independent factors influence convergent thinking are alike, as both bilingualism and cortical thinness in SMA facilitate convergent thinking by enhancing this selective ability. Taking into account the study mentioned above, which reported greater GMV in right SMA in early bilingual children than monolingual children (Schug et al., 2022), the relations among properties of bilingualism, morphometric measures of SMA and convergent thinking should be investigated further, as they still possibly interact with each other.

There are several limitations in the present study. First, as a cross sectional study, results on the developmental patterns observed must be interpreted with caution. As mentioned above, a longitudinal study would be useful to determine more conclusively how the effect of bilingualism on convergent thinking changes with age. Second, the sample size was relatively small. With only 7 adolescents, we could not compare children and adolescents, which future studies with larger populations could do so as to highlight any developmental differences which may potentially emerge between these groups. Third, this study did not find a structural basis of the effect of bilingualism on convergent thinking. If bilingualism is not long enough to change the structures of brains across development, it may be worth exploring instead whether bilingualism may change the functional activation in some brain regions. An fMRI study on this topic would allow addressing this point. Also, future work should explore creative thinking with different tasks (i.e., verbal tasks), as the drawing-based task may hinder/affect the results observed. In fact, given the human-based subjective rating of the creative outcomes, our measures may not capture individual creativity properly, or favor individuals with higher sensori-motor skills. Finally, the reason for the thinning of the cortex in right SMA is unclear. It is recommended that more details about the bilingual experience, like age of acquisition of the second language and language proficiency, be taken into account in future studies, as these properties of bilingualism might be the potential factors in modulating the brain structures (Schug et al., 2022).

## 5. Conclusion

In summary, this study indicates that convergent thinking, but not divergent thinking benefits from bilingualism in children and adolescents. However, this bilingual advantage appears to weaken across development. In addition, the morphometric analysis showed that convergent thinking ability was negatively correlated with the cortical thickness in the right SMA. Bilingualism and cortical thinness in right SMA might be two independent beneficial factors for convergent thinking.

## Data availability statement

The raw data supporting the conclusions of this article will be made available by the authors, without undue reservation.

## Ethics statement

The studies involving human participants were reviewed and approved by the Local Ethics Committee (CER-Vaud). Written informed consent to participate in this study was provided by the participants or their legal guardian/next of kin.

## Author contributions

YZ performed the morphometric analyses of the MRI data and wrote the manuscript. SDe designed the experiment, collected the data, performed the statistical analyses of demographic and neuropsychological data, and revised the manuscript. SDu conceived of the study and its research questions, coordinated the collaboration, and revised the manuscript. All authors contributed to the article and approved the submitted version.

## Footnotes

1 It is important to specify that the drawing-based tasks described here are not rated on the “beauty” nor the “figurative” component of the drawing. What is assessed is the capacity of the child to combine the shapes (for the convergent thinking task), or to derive new ideas (for the divergent thinking task).

2
http://dbm.neuro.uni-jena.de/cat12/

3
http://www.fil.ion.ucl.ac.uk/spm/software/spm12/
