# Supplementary material for: Bilingualism and creativity across development: Evidence from divergent thinking and convergent thinking
Source: Front Hum Neurosci. 2023 Jan 6;16:1058803. doi: 10.3389/fnhum.2022.1058803 (PMC9859714; doi:10.3389/fnhum.2022.1058803)
Supplement: Supplementary file 1 [file Table_1.docx]

Table S1: ANCOVA on the convergent thinking scores on the subset of the 67 participants presenting both creativity measures.

| ANCOVA - Convergent score | | | | | | | | | | | |  |
| --- | --- | --- | --- | --- | --- | --- | --- | --- | --- | --- | --- | --- |
|  | | **Sum of Squares** | | **df** | | **Mean Square** | | **F** | | **p** | |  |
| Bilingualism |  | 25.00123 |  | 1 |  | 25.00123 |  | 10.24398 |  | 0.002***** |  | |
| Age |  | 0.00498 |  | 1 |  | 0.00498 |  | 0.00204 |  | 0.964 |  | |
| Pedagogy |  | 0.41217 |  | 1 |  | 0.41217 |  | 0.16888 |  | 0.683 |  | |
| SES |  | 1.77050 |  | 1 |  | 1.77050 |  | 0.72544 |  | 0.398 |  | |
| PM47 |  | 0.39799 |  | 1 |  | 0.39799 |  | 0.16307 |  | 0.688 |  | |
| Bilingualism ✻ Pedagogy |  | 0.28711 |  | 1 |  | 0.28711 |  | 0.11764 |  | 0.733 |  | |
| Bilingualism ✻ Age |  | 25.73899 |  | 1 |  | 25.73899 |  | 10.54627 |  | 0.002***** |  | |
| Pedagogy ✻ Age |  | 1.29885 |  | 1 |  | 1.29885 |  | 0.53219 |  | 0.469 |  | |
| Residuals |  | 139.11289 |  | 57 |  | 2.44058 |  |  |  |  |  | |
|  | | | | | | | | | | | |  |
